# Are bacterial infections a major cause of cardiovascular disease?

**DOI:** 10.3389/fcvm.2024.1389109

**Published:** 2024-09-04

**Authors:** Dohn Kissinger

**Affiliations:** Independent Researcher, El Dorado Hills, CA, United States

**Keywords:** cardiovascular disease, statin drugs, cholesterol, LDL-C, HDL-C

## Introduction

Much of the current effort to treat cardiovascular disease (CVD) involves reducing the level of low-density lipoprotein cholesterol (LDL-C), which is also known as the “bad cholesterol.” LDL-C is however not a cholesterol; it is a lipoprotein that transports cholesterol from the liver to repair damaged tissues in the human body. The high-density lipoprotein cholesterol (HDL-C), known as the “good cholesterol,” returns the unused cholesterol to the liver. We should consider LDL-C and HDL-C as essential components of the natural cholesterol cycle that provide cholesterol to help heal damaged bodily tissues and return the unused cholesterol to the liver.

## Discussion

Issues with the cholesterol theory include the following:

• Reducing cholesterol levels to low values leads to low levels of serum serotonin, increasing the risk of depression and suicide (1). This is contrary to the cholesterol theory, which assumes that lower cholesterol levels are preferable.
• Most people over 60 years of age have lower mortality rates with higher LDL-C levels (2).
• A large clinical trial (3) that used the medication evacetrapib was performed to show that decreasing LDL-C and increasing HDL-C would help protect patients from major cardiovascular events such as heart attack and stroke. Although the LDL-C was decreased by an average of 37% and the HDL-C was increased by an average of 130% compared to those in patients taking a placebo, the medication did not improve cardiovascular health. The results of this trial disprove the cholesterol theory.
• A recent article analyzed all-cause mortality rates as a function of LDL-C values (4). The lowest all-cause mortality rate was for LDL-C levels in the range of 160–189.9. Lowering cholesterol from this level to a low LDL-C level in the range of 70–99.9 resulted in an approximately 20% increase in the all-cause mortality rate. Lowering cholesterol to a very low cholesterol level with LDL-C below 70 resulted in over a 50% increase in the all-cause mortality rate.

All of these examples show that reduced LDL-C levels have either equal or higher mortality rates compared with high LDL-C levels. This contradicts the cholesterol theory, which assumes that lower LDL-C values imply lower mortality rates. We therefore conclude that high LDL-C levels are not a cause of CVD.

Proponents of statin drug therapy may state that statin drugs are effective in treating CVD. This may be due to the antibacterial and anti-inflammatory properties (5, 6) of statin drugs rather than the reduction of LDL-C levels. However, if higher cholesterol levels are beneficial, then decreasing cholesterol levels with statin drugs is counterproductive. It would be preferable to use antibacterial medications that do not decrease cholesterol levels.

If high levels of LDL-C are not the cause of CVD, then what is? An article published in 2012 (7) postulated that bacterial infections could be a cause of CVD. Ever since, evidence has accumulated to validate this theory. As discussed in a Harvard Health article (8), there is a connection between bacterial gum disease and heart disease. There also seems to be a relationship between some gut microbiota and heart disease (9). In addition, a study from the University of Minnesota (10) concludes that “CVD patients had higher odds of infection within 90 days preceding their CVD event compared with equivalent control periods 1 and 2 years previous.”

What is the mechanism by which a bacterial infection can injure the arteries? Peptic ulcers develop because *Helicobacter* *pylori* bacteria eat away the protective lining of the digestive system. Just as occurs with ulcers, a bacterial infection may eat away at the walls of the arteries. Some research has been performed to confirm this theory (11–15).

A review published in 2023 (16) summarizes the status of the connection between bacterial infections and CVD. This review contains many additional references that show that bacterial infections are a major cause of CVD. It concludes that “Abundant evidence supports the view that infectious diseases are a major cause of atherosclerosis, AMI and myocarditis and that inflammation is a protective reaction against infection, rather than the very cause. As bacteremia and sepsis have been found in many patients with serious AMI, we suggest that blood cultures should be performed in all patients with AMI or stroke, and if positive, the patient should be treated with a suitable antibiotic.”

## Conclusions

It took decades for the medical community to accept that bacterial infections are a major cause of peptic ulcers. This revolutionized the treatment of peptic ulcers. After the medical community accepts that bacterial infections are a major cause of CVD, the treatment of CVD will be similarly revolutionized.
